# Supplementary material for: Performance of Commercial Dermatoscopic Systems That Incorporate Artificial Intelligence for the Identification of Melanoma in General Practice: A Systematic Review
Source: Cancers (Basel). 2024 Apr 8;16(7):1443. doi: 10.3390/cancers16071443 (PMC11011068; doi:10.3390/cancers16071443)
Supplement: Supplementary file 1 [file cancers-16-01443-s001.zip › cancers-2917526-supplementary.pdf]

SUPPLEMENT 1

Search strategy, inclusion and exclusion criteria, search string and captured articles

Topic:

Performance of market approved, Artificial Intelligence for the Classification of Melanoma in Practice

Study Questions

What is the diagnostic accuracy of artificial intelligence in detecting MM in practice?

Inclusion and Exclusion Criteria

Inclusion Criteria

Empirical research reports published in English in peer-reviewed scientific journals between 1st January 2018 and 31st December 2023 that answer the research question.

Systematic reviews were included in the search to identify model studies, and to check the search terms captured these studies.

Exclusion Criteria

We will exclude articles that:

- provide insufficient data to answer the research question
- failed to use systematic research design and reporting procedures
- are in the form of commentary, letter to the editor, reviews, editorials, thesis, government and non-governmental reports
- Outside years 2018 through to 2024
- are not published in English

Search Strategy

Databases: Five databases: CINAHL (EBSCOHost), Medline (EBSCOHost), ScienceDirect, Scopus and Web of Science.

Supplement Table S1: Search string

**Characteristics**	**MeSH Search Terms**
**Population**	melanoma OR "cutaneous melanoma" OR nevi OR "pigmented lesion" OR "malignant melanoma"
**Intervention**	artificial intelligence OR "machine learning" OR "neural network" OR "deep learning"
**Control**	dermoscopy OR dermatology OR dermatoscopy OR telederm* OR image* OR "skin screen*" OR "skin exam*" OR "skin clinic" OR "skin cancer screening" OR "mobile application*" OR "mobile skin screen*" OR "mobile skin cancer screen*" OR "primary care" OR "secondary care" OR "tertiary care" OR "health screen*" OR "cancer screen*"
**Outcome**	"diagnostic accuracy" OR (sensitivity AND specificity) OR class* OR detect* OR diagnos*

CINAHL : (melanoma OR "cutaneous melanoma" OR nevi OR "pigmented lesion" OR "malignant melanoma") AND ("diagnostic accuracy" OR (sensitivity AND specificity) OR class* OR detect* OR diagnos*) AND (artificial intelligence OR "machine learning" OR "neural network" OR "deep learning") AND (dermoscopy OR dermatology OR dermatoscopy OR telederm* OR image* OR "skin screen*" OR "skin exam*" OR "skin clinic" OR "skin cancer screening" OR "mobile application*" OR "mobile skin screen*" OR "mobile skin cancer screen*" OR "primary care" OR "secondary care" OR "tertiary care" OR "health screen*" OR "cancer screen*")

Add: Date Filter between 01/01/2018 to present AND English language only

Supplement Table S2: CINAHL keywords and captured articles

**Search**	**Term**	**Keywords**	**Date of search (22 Dec 2023)**	**Date of search (07 Jan 2024**
**1**	**All Fields**	melanoma OR "cutaneous melanoma" OR nevi OR "pigmented lesion" OR "malignant melanoma"	22,343	22,343
**2**	**All Fields**	"diagnostic accuracy" OR (sensitivity AND specificity) OR class* OR detect* OR diagnos*	1,756,789	1,758,042
**3**	**All Fields**	artificial intelligence OR "machine learning" OR "neural network" OR "deep learning"	35,776	35,988
**4**	**All Fields**	dermoscopy OR dermatology OR dermatoscopy OR telederm* OR image* OR "skin screen*" OR "skin exam*" OR "skin clinic" OR "skin cancer screening" OR "mobile application*" OR "mobile skin screen*" OR "mobile skin cancer screen*" OR "primary care" OR "secondary care" OR "tertiary care" OR "health screen*" OR "cancer screen*"	394,847	395,103
**Combined search with AND (1,2,3,4)**			122	122
**Filter date (01-01-2018 to 31-12-2023)**			113	113
**English language only**			113	113*

Note: * No additional articles captured

Medline: (melanoma OR "cutaneous melanoma" OR nevi OR "pigmented lesion" OR "malignant melanoma") AND ("diagnostic accuracy" OR (sensitivity AND specificity) OR class* OR detect* OR diagnos*) AND (artificial intelligence OR "machine learning" OR "neural network" OR "deep learning") AND (dermoscopy OR dermatology OR dermatoscopy OR telederm* OR image* OR "skin screen*" OR "skin exam*" OR "skin clinic" OR "skin cancer screening" OR "mobile application*" OR "mobile skin screen*" OR "mobile skin cancer screen*" OR "primary care" OR "secondary care" OR "tertiary care" OR "health screen*" OR "cancer screen*")

Add: Date Filter between 01/01/2018 to present AND English language only

Supplement Table S3: Medline keywords and captured articles

**Search**	**Term**	**Keywords**	**n = Records captured; Date of search (08 Nov 2023)**	**n = Records captured; Date of search (07 Jan 2024**
**1**	**All Fields**	melanoma OR "cutaneous melanoma" OR nevi OR "pigmented lesion" OR "malignant melanoma"	170,123	171,482
**2**	**All Fields**	"diagnostic accuracy" OR (sensitivity AND specificity) OR class* OR detect* OR diagnos*	9,953,917	10,033,330
**3**	**All Fields**	artificial intelligence OR "machine learning" OR "neural network" OR "deep learning"	255,020	265,582
**4**	**All Fields**	dermoscopy OR dermatology OR dermatoscopy OR telederm* OR image* OR "skin screen*" OR "skin exam*" OR "skin clinic" OR "skin cancer screening" OR "mobile application*" OR "mobile skin screen*" OR "mobile skin cancer screen*" OR "primary care" OR "secondary care" OR "tertiary care" OR "health screen*" OR "cancer screen*"	1,685,425	1,703,632
**Combined search with AND (1,2,3,4)**			768	812
**Filter date (01-01-2018 to 31-12-2023)**			645	687
**English language only**			634	676*

Note: * 42 additional articles captured

ScienceDirect: melanoma AND "artificial intelligence" AND detection AND accuracy AND clinic AND dermatology

Add: Date Filter between 01/01/2018 to present AND English language only

Supplement Table S4: ScienceDirect keywords and captured articles

**Search**	**Term**	**Keywords**	**n = Records captured; Date of search (07 Jan 2024**
**1**	**All Fields**	melanoma AND "artificial intelligence" AND detection AND accuracy AND clinic AND dermatology	195
**Filter date (01-01-2018 to 31-12-2023) AND English language only**			155
**Review articles AND Research articles**			101

Scopus: (TITLE-ABS-KEY (melanoma OR "cutaneous melanoma" OR nevi OR "pigmented lesion" OR "malignant melanoma")) AND (TITLE-ABS-KEY ("diagnostic accuracy" OR (sensitivity AND specificity) OR class* OR detect* OR diagnos*)) AND (TITLE-ABS-KEY (artificial AND intelligence OR "machine learning" OR "neural network" OR "deep learning")) AND (TITLE-ABS-KEY (dermoscopy OR dermatology OR dermatoscopy OR telederm* OR image* OR "skin screen*" OR "skin exam*" OR "skin clinic" OR "skin cancer screening" OR "mobile application*" OR "mobile skin screen*" OR "mobile skin cancer screen*" OR "primary care" OR "secondary care" OR "tertiary care" OR "health screen*" OR "cancer screen*")) AND PUBYEAR > 2017 AND PUBYEAR < 2024 AND (LIMIT-TO (LANGUAGE , "English")) AND (LIMIT-TO (DOCTYPE , "ar") OR LIMIT-TO (DOCTYPE , "re"))

Supplement Table S5: Scopus keywords and captured articles

**Search**	**Term**	**Keywords**	**n = Records captured; Date of search (08 Nov 2023)**	**n = Records captured; Date of search (07 Jan 2024**
**1**	**Article title, Abstract, Keywords**	melanoma OR "cutaneous melanoma" OR nevi OR "pigmented lesion" OR "malignant melanoma"	248,403	250,602
**2**	**Article title, Abstract, Keywords**	"diagnostic accuracy" OR (sensitivity AND specificity) OR class* OR detect* OR diagnos*	16,458,756	16,634,188
**3**	**Article title, Abstract, Keywords**	artificial intelligence OR "machine learning" OR "neural network" OR "deep learning"	728,154	746,053
**4**	**Article title, Abstract, Keywords**	dermoscopy OR dermatology OR dermatoscopy OR telederm* OR image* OR "skin screen*" OR "skin exam*" OR "skin clinic" OR "skin cancer screening" OR "mobile application*" OR "mobile skin screen*" OR "mobile skin cancer screen*" OR "primary care" OR "secondary care" OR "tertiary care" OR "health screen*" OR "cancer screen*"	3,864,952	3,916,976
**Combined search with AND (1,2,3,4)**			1,003	1,053
**Filter date (01-01-2018 to 31-12-2023)**			757	799
**English language only**			731	773
**Limited to Article AND Review**			516	545*

Note: * 29 additional articles captured

Web of Science

melanoma OR "cutaneous melanoma" OR nevi OR "pigmented lesion" OR "malignant melanoma" (Topic) AND "diagnostic accuracy" OR (sensitivity AND specificity) OR class* OR detect* OR diagnos* (Topic) AND artificial intelligence OR "machine learning" OR "neural network" OR "deep learning" (Topic) AND dermoscopy OR dermatology OR dermatoscopy OR telederm* OR image* OR "skin screen*" OR "skin exam*" OR "skin clinic" OR "skin cancer screening" OR "mobile application*" OR "mobile skin screen*" OR "mobile skin cancer screen*" OR "primary care" OR "secondary care" OR "tertiary care" OR "health screen*" OR "cancer screen*" (Topic) and Preprint Citation Index (Exclude – Database) and English (Languages) and Article or Review Article (Document Types) AND date filter 2018-2013.

Supplement Table S6: Web of science keywords and captured articles

**Search**	**Term**	**Keywords**	**n = Records captured; Date of search (08 Nov 2023)**	**n = Records captured; Date of search (07 Jan 2024**
**1**	**Topic**	melanoma OR "cutaneous melanoma" OR nevi OR "pigmented lesion" OR "malignant melanoma"	290,123	293,098
**2**	**Topic**	"diagnostic accuracy" OR (sensitivity AND specificity) OR class* OR detect* OR diagnos*	21,293,654	21,476,299
**3**	**Topic**	artificial intelligence OR "machine learning" OR "neural network" OR "deep learning"	1,381,654	1,462,195
**4**	**Topic**	dermoscopy OR dermatology OR dermatoscopy OR telederm* OR image* OR "skin screen*" OR "skin exam*" OR "skin clinic" OR "skin cancer screening" OR "mobile application*" OR "mobile skin screen*" OR "mobile skin cancer screen*" OR "primary care" OR "secondary care" OR "tertiary care" OR "health screen*" OR "cancer screen*"	4,151,086	4,194,396
**Combined search with AND (1,2,3,4)**			2,086	2,167
**Filter date (01-01-2018 to 31-12-2023)**			1,712	1,838
**English language only**			1,680	1,802
**Limit to Article AND review**			1,284	1,337*

Note: *53 additional records identified

SUPPLEMENT 2

Supplementary Table S7. Critical appraisal of included studies

Author, Year, Country	Study Design	Level of Evidence*	Modified Axis (Average for reviewers)	Percentage (%)	Quality Rating
Anderson et al., 2023, USA [34]	Cross-sectional study	III-3	13/20	65.0	Fair
Cerminara et al., 2023, Switzerland [37]	Prospective, single-center, observational study	III-2	17/20	85.0	Good
Fink et al., 2020, Germany [43]	Cross-Sectional Study	III-2	17/20	85.0	Good
Jahn et al., 2022, Switzerland [35]	Prospective, single-center, comparative observational cohort study	III-2	17/20	85.0	Good
MacLellan et al., 2021, Canada [44]	Prospective diagnostic accuracy study	III-2	16.5/20	82.5	Good
Marchetti et al., 2023, USA [38]	Single-center, retrospective, observational convenience study	III-2	17/20	85.0	Good
Martin-Gonzales et al., 2022, Spain [45]	Retrospective cross-sectional study	III-2	17/20	85.0	Good
Menzies et al., 2023, Australia [46]	Multicenter, prospective, diagnostic, clinical trial	II	19/20	95.0	Good
Miller et al., 2023, Australia [47]	Cross-Sectional study	III-1	17/20	85.0	Good
Phillips, 2019, United Kingdom [48]	Prospective, multicenter, single-arm, masked diagnostic trial	III-1	16/20	80.0	Good

Thomas et al., 2023, United Kingdom [49]	Prospective, Multicenter, diagnostic clinical trial	II	16.5/20	82.5	Good
Udrea et al., 2020, Romania/ Netherlands [36]	Retrospective, diagnostic, clinical trail	III-2	17/20	85.0	Good
Winkler et al., 2023, Germany [39]	Multicenter Prospective, diagnostic study	III-1	17/20	85.0	Good
Winkler et al, 2019, Germany [40]	Retrospective Cross-sectional analysis	III-3	17/20	85.0	Good
Winkler et al., 2021, Germany [41]	Retrospective Cross-sectional analysis	III-3	16/20	80.0	Good
Winkler et al., 2022, Germany [42]	Retrospective cross-sectional study	III-2	17/20	85.0	Good
			k = 0.977	Mean=83.4% SD±5.9	

Where: * Level of Evidence [32], k = Cohen's kappa score, SD = standard deviation

References*

* (as numbered in the main manuscript)

32. National Health and Medical Research Council. NHMRC Evidence Hierarchy: designations of 'levels of evidence' according to type of research questions. 2009. Available online: https://www.nhmrc.gov.au/ (accessed on 29 January 2024).

34. Anderson JM, Tejani I, Jarmain T, Kellett L, Moy RL. Artificial Intelligence vs Medical Providers in the Dermoscopic Diagnosis of Melanoma. Cutis. 2023;111(5):254-8.

35. Jahn AS, Navarini AA, Cerminara SE, Kostner L, Huber SM, Kunz M, Maul JT, Dummer R, Sommer S, Neuner AD, Levesque MP, Cheng PF, Maul LV. Over-Detection of Melanoma-Suspect Lesions by a CE-Certified Smartphone App: Performance in Comparison to Dermatologists, 2D and 3D Convolutional Neural Networks in a Prospective Data Set of 1204 Pigmented Skin Lesions Involving Patients' Perception. Cancers. 2022;14(15).

36. Udrea A, Mitra GD, Costea D, Noels EC, Wakkee M, Siegel DM, de Carvalho TM, Nijsten TEC. Accuracy of a smartphone application for triage of skin lesions based on machine learning algorithms. Journal of the European Academy of Dermatology and Venereology : JEADV. 2020;34(3):648-55.

37. Cerminara SE, Cheng P, Kostner L, Huber S, Kunz M, Maul JT, Böhm JS, Dettwiler CF, Geser A, Jakopović C, Stoffel LM, Peter JK, Levesque M, Navarini AA, Maul LV. Diagnostic performance of augmented intelligence with 2D and 3D total body photography and convolutional neural networks in a high-risk population for melanoma under real-world conditions: A new era of skin cancer screening? Eur J Cancer. 2023;190.

38. Marchetti MA, Nazir ZH, Nanda JK, Dusza SW, D'Alessandro BM, DeFazio J, Halpern AC, Rotemberg VM, Marghoob AA. 3D Whole-body skin imaging for automated melanoma detection. J Eur Acad Dermatol Venereol. 2023;37(5):945-50.

39. Winkler JK, Blum A, Kommoss K, Enk A, Toberer F, Rosenberger A, Haenssle HA. Assessment of Diagnostic Performance of Dermatologists Cooperating with a Convolutional Neural Network in a Prospective Clinical Study: Human with Machine. JAMA Dermatology. 2023;159(6):621-7.

40. Winkler JK, Fink C, Toberer F, Enk A, Deinlein T, Hofmann-Wellenhof R, Thomas L, Lallas A, Blum A, Stolz W, Haenssle HA. Association Between Surgical Skin Markings in Dermoscopic Images and Diagnostic Performance of a Deep Learning Convolutional Neural Network for Melanoma Recognition. JAMA dermatology. 2019;155(10):1135-41.

41. Winkler JK, Sies K, Fink C, Toberer F, Enk A, Abassi MS, Fuchs T, Haenssle HA. Association between different scale bars in dermoscopic images and diagnostic performance of a market-approved deep learning convolutional neural network for melanoma recognition. European journal of cancer (Oxford, England : 1990). 2021;145:146-54.

42. Winkler JK, Tschandl P, Toberer F, Sies K, Fink C, Enk A, Kittler H, Haenssle HA. Monitoring patients at risk for melanoma: May convolutional neural networks replace the strategy of sequential digital dermoscopy? European journal of cancer (Oxford, England : 1990). 2022;160:180-8.

43. Fink C, Blum A, Buhl T, Mitteldorf C, Hofmann-Wellenhof R, Deinlein T, Stolz W, Trennheuser L, Cussigh C, Deltgen D, Winkler JK, Toberer F, Enk A, Rosenberger A, Haenssle HA. Diagnostic performance of a deep learning convolutional neural network in the differentiation of combined naevi and melanomas. Journal of the European Academy of Dermatology and Venereology : JEADV. 2020;34(6):1355-61.

44. MacLellan AN, Price EL, Publicover-Brouwer P, Matheson K, Ly TY, Pasternak S, Walsh NM, Gallant CJ, Oakley A, Hull PR, Langley RG. The use of noninvasive imaging techniques in the diagnosis of melanoma: a prospective diagnostic accuracy study. Journal of the American Academy of Dermatology. 2021;85(2):353-9.

45. Martin-Gonzalez M, Azcarraga C, Martin-Gil A, Carpena-Torres C, Jaen P. Efficacy of a Deep Learning Convolutional Neural Network System for Melanoma Diagnosis in a Hospital Population. International Journal of Environmental Research and Public Health. 2022;19(7).

46. Menzies SW, Sinz C, Menzies M, Lo SN, Yolland W, Lingohr J, Razmara M, Tschandl P, Guitera P, Scolyer RA, Boltz F, Borik-Heil L, Herbert Chan H, Chromy D, Coker DJ, Collgros H, Eghtedari M, Corral Forteza M, Forward E, Gallo B, Geisler S, Gibson M, Hampel A, Ho G, Junez L, Kienzl P, Martin A, Moloney FJ, Regio Pereira A, Ressler JM, Richter S, Silic K, Silly T, Skoll M, Tittes J, Weber P, Weninger W, Weiss D, Woo-Sampson P, Zilberg C, Kittler H. Comparison of humans versus mobile phone-powered artificial intelligence for the diagnosis and management of pigmented skin cancer in secondary care: a multicentre, prospective, diagnostic, clinical trial. Lancet Digit Heal. 2023;5(10):e679-e91.

47. Miller IJ, Stapelberg M, Rosic N, Hudson J, Coxon P, Furness J, Walsh J, Climstein M. Implementation of artificial intelligence for the detection of cutaneous melanoma within a primary care setting: prevalence and types of skin cancer in outdoor enthusiasts. PeerJ. 2023;11.

48. Phillips M, Marsden H, Jaffe W, Matin RN, Wali GN, Greenhalgh J, McGrath E, James R, Ladoyanni E, Bewley A, Argenziano G, Palamaras I. Assessment of Accuracy of an Artificial Intelligence Algorithm to Detect Melanoma in Images of Skin Lesions. JAMA Network Open. 2019;2(10).

49. Thomas L, Hyde C, Mullarkey D, Greenhalgh J, Kalsi D, Ko J. Real-world post-deployment performance of a novel machine learning-based digital health technology for skin lesion assessment and suggestions for post-market surveillance. Frontiers in medicine. 2023;10:1264846.
